# Editorial: Evolution in respiratory pharmacology

**DOI:** 10.3389/fphar.2023.1329811

**Published:** 2023-11-17

**Authors:** Barbara Ruaro, Riccardo Pozzan, Alessia Giovanna Andrisano, Marco Confalonieri, Nilesh Sudhakar Ambhore

**Affiliations:** ^1^ Pulmonology Unit, Department of Medical Surgical and Healt Sciencies, Hospital of Cattinara, University of Trieste, Trieste, Italy; ^2^ Pulmonology Unit, Azienda Ospedaliera Santa Maria degli Angeli, Pordenone, Italy; ^3^ Pulmonology Unit, Ospedale Civile di Gorizia, Gorizia, Italy; ^4^ Department of Pharmaceutical Sciences, School of Pharmacy, College of Health and Human Sciences, North Dakota State University, Fargo, ND, United States

**Keywords:** respiratory medicine, lung diseases, respiratory pharmacology, airway inflammation, airway dysfunction

This collection of Research Topics entitled “*Evolution in respiratory pharmacology*,*”* involving authors from several countries, confirms that “Respiratory Pharmacology” is a current topic in clinical and research settings.

All articles focused on contributions that explore the changing context and emerging new perspectives within Respiratory Pharmacology (Kim et al., Cerqua et al., Li et al., Lin et al., Zhang et al.). The emphasis of this Research Topic is on the dynamics of change and the evolution of the latest progress made in the field of Respiratory Pharmacology. This collection of articles aims to inform, inspire, and provide direction and guidance to researchers in the field.

In the first article, the authors evaluated whether vegetable glycerin (VG) e-cigarette (e-cig) aerosols, in the absence of nicotine and flavors, impact parameters of mucociliary function in human volunteers, a large animal model (sheep), and air-liquid interface (ALI) cultures of primary human bronchial epithelial cells (HBECs) (Kim et al.). It is important to underline that the VG and propylene glycol (PG) serve as delivery vehicles for nicotine and flavorings in most e-cig liquids. Interestingly, the authors found that VG-containing e-cig aerosols reduced the activity of nasal cystic fibrosis transmembrane conductance regulator (CFTR) in human volunteers who vaped for 7 days. The results of this study show that VG e-cig aerosols did not increase matrix metalloproteinase-9 (MMP9) mRNA expression. On the contrary, the authors observed a significant increase of the expression levels of interleukin-6 (IL-6), interleukin-8 (IL-8), transforming growth factor-beta 1 (TGF-β1) and mucin 5AC (MUC5AC) mRNAs in HBECs after 7 days of VG e-cig aerosols exposure. The researchers’ conclusions are that VG e-cig aerosols can potentially induce airway inflammation and ion channel dysfunction with consequent mucus hyper-concentration (Kim et al.).

The second study aimed to evaluate if artesunate inhibits airway remodeling in asthma via the MAPK signaling pathway (Zhang et al.). Artesunate (ART) is a semi-synthetic water-soluble artemisinin derivative extracted from the plant Artemisia annua. Previous *in vivo* and *in vitro* studies using Artemisia annua suggest it may help to decrease inflammation and attenuate airway remodeling in asthma. However, its mechanism of action is not clear. To clarify underlying mechanisms, in an experimental animal model of asthma *in vivo*, female BALB/c mice were sensitized them via ovalbumin (OVA) to establish the asthma model, followed by carrying out ART interventions. Kyoto Encyclopedia of Genes and Genomes (KEGG) pathway analysis revealed that the ART played a protective role via various pathways including the mitogen-activated protein kinase (MAPK) pathway. Moreover, ART could reduce the overexpression in inflammatory zone 1 (FIZZ1) as revealed by immunohistochemistry (IHC) and Western blot analyses. ART attenuated OVA-induced asthma by downregulating phosphorylated p38 MAPK. ART considerably attenuated inflammatory cell infiltration, mucus secretion, and collagen fibers deposition. In conclusion, the authors reported that ART exerted multitarget and multi-pathway protective effects in an experimental animal model of asthma which require further research. FIZZ1 was identified as a potential target for asthma airway remodeling, targeting the MAPK pathway was a key mechanism for ART-induced protective effects against asthmatic-like changes (Zhang et al.).

The third study reported the results of a multicenter, prospective observational study (Lin et al.). The authors compared of treatment persistence, adherence, and risk of exacerbation in patients with COPD treated with single-inhaler triple therapy (SITT) *versus* multiple-inhaler triple therapy (MITT) in the Chinese population. A total of 1,328 patients were enrolled, including 535 (40.3%) patients treated with SITT and 793 (59.7%) treated with MITT. All patients were followed up for 1 year. SITT patients had a significantly higher rate of adherence and treatment persistence. Conversely, they had a significantly lower risk of moderate-to-severe and severe exacerbation and also a reduced risk of all-cause mortality compared to MITT patients (Lin et al.).

The fourth article was a case report of a 17-year-old boy who underwent lung transplantation (LT) for the treatment of paraquat acute toxicity, along with a summary of the postoperative complications (Li et al.). The authors also conducted an interesting literature review. The researchers identified eight case reports involving 11 patients with paraquat poisoning who underwent LT. Three patients died due to paraquat poisoning, which led to fibrosis in the transplanted lungs or postoperative complications, while eight patients survived during the follow-up. In summary, the authors concluded that LT should be planned when hepatorenal function starts to recover, and waiting for complete recovery is unnecessary. Prevention of infection before surgery is important to reduce the incidence of postoperative infection. A multidisciplinary team can successfully manage complex perioperative complications caused by the herbicide itself or the late timing of transplantation (Li et al.).

In the last research study, the authors evaluated whether the prednisone-hydrogen sulfide-releasing hybrid shows an improved therapeutic profile in asthma (Cerqua et al.). Hydrogen sulfide (H2S) is emerging as an important potential therapeutic option for respiratory inflammatory diseases. The authors investigated the effectiveness of a novel corticosteroid derivative, that is chemically linked to an H2S donor, in managing asthma features. The effects of prednisone (PS), the H2S donor (4-hydroxybenzamide; TBZ), and their combination (PS-TBZ) have been evaluated *in vitro* and *in vivo*. The *in vitro* experiments were conducted using lipopolysaccharide stimulated J774 macrophages, while the *in vivo* experiments utilized an experimental asthma model (Cerqua et al.). In the *in vitro* study, PS-TBZ exhibited an increased effect compared to the individual parent compounds in modulating the production of inflammatory mediators. TBZ also significantly reduced bronchial contractility and enhanced bronchial relaxation (Cerqua et al.). The *in vivo* experiments were conducted by administering the PS, TBZ, or PS-TBZ to ovalbumin-sensitized BALB/c mice. BALB/c mice were sensitized with a subcutaneous administration of ovalbumin (OVA, 100 µg) complexed with alum (13 mg/mL) on days 0 and 7 (Cerqua et al.). On the sixth and seventh days animal groups were pretreated intraperitoneally 30 min before OVA with PS (1 mg/kg, 60 nmol), TBZ (130 nmol), and PS-TBZ (30 nmol). This treatment has been protracted every day up to the 14th day of sensitization. The obtained results confirmed that PS-TBZ had a significantly better action in controlling airway hyperreactivity as compared to TBZ or PS alone. Moreover, PS-TBZ was more effective in restoring salbutamol-induced relaxation. Immunohistochemistry analysis showed a significant reduction in the production of α-SMA and procollagen III, indicating the efficacy of PS-TBZ in controlling airway remodeling. Moreover, PS-TBZ also promoted epithelial repair recovery of the bronchial and parenchyma structure with mucin production inhibition. This study suggests that PS-TBZ co-formulation deserves further research as it might provide a new potential pharmacological strategy to improve the therapeutic effects of glucocorticoids in asthma (Cerqua et al.).

In conclusion, this Research Topic pays particular attention to recent progress made in the use of innovative treatments, which is expected to provide new insights into research (Kim et al., Cerqua et al., Li et al., Lin et al., Zhang et al.). Our poor understanding of the underlying causes motivates growing interest in the lungs and their diseases to find new treatment regimens to induce complete recovery of these patients (Baratella et al., 2021; Calzetta et al., 2021; Cazzola et al., 2021; Matera et al., 2021; Confalonieri et al., 2022). The presentation of the latest cutting-edge approaches to the treatment of lung diseases has potential implications for opening new avenues and changing the medical practise in the near future.
